# Primary care blood tests before cancer diagnosis: National Cancer Diagnosis Audit data

**DOI:** 10.3399/BJGP.2022.0265

**Published:** 2022-10-18

**Authors:** Ben M Cranfield, Minjoung Monica Koo, Gary A Abel, Ruth Swann, Sean McPhail, Greg P Rubin, Georgios Lyratzopoulos

**Affiliations:** University College London, London.; University College London, London.; University of Exeter Medical School, St Luke’s Campus, Exeter.; National Disease Registration Service, NHS Digital, Leeds, and Cancer Research UK, London.; National Disease Registration Service, NHS Digital, Leeds.; Population Health Sciences Institute, Newcastle University, Newcastle Upon Tyne.; University College London, London.

**Keywords:** blood tests, cancer, cancer diagnosis, full blood count, liver function tests, primary health care, urea and electrolytes

## Abstract

**Background:**

Blood tests can support the diagnostic process in patients with cancer but how often they are used is unclear.

**Aim:**

To explore use of common blood tests before cancer diagnosis in primary care.

**Design and setting:**

English National Cancer Diagnosis Audit data on 39 752 patients with cancer diagnosed in 2018.

**Method:**

Common blood test use (full blood count [FBC], urea and electrolytes [U&E], and liver function tests [LFTs]), variation by patient and symptom group, and associations with the primary care interval and the diagnostic interval were assessed.

**Results:**

At least one common blood test was used in 41% (*n* = 16 427/39 752) of patients subsequently diagnosed with cancer. Among tested patients, (*n* = 16 427), FBC was used in 95% (*n* = 15 540), U&E in 89% (*n* = 14 555), and LFTs in 76% (*n* = 12 414). Blood testing was less common in females (adjusted odds ratio versus males: 0.92, 95% confidence interval [CI] = 0.87 to 0.98) and Black and minority ethnic patients (0.89, 95% CI = 0.82 to 0.97 versus White), and more common in older patients (1.12, 95% CI = 1.06 to 1.18 for ≥70 years versus 50–69 years). Test use varied greatly by cancer site (melanoma 2% [ *n* = 55/2297]; leukaemia 84% [ *n* = 552/661]). Fewer patients presenting with alarm symptoms alone were tested (24% [ *n* = 3341/13 778]) than those with non-alarm symptoms alone (50% [ *n* = 8223/16 487]). Median primary care interval and diagnostic interval were longer in tested than non-tested patients (primary care interval: 10 versus 0 days; diagnostic interval: 49 versus 32 days, respectively, *P*<0.001 for both), including among tested patients with alarm symptoms (primary care interval: 4 versus 0 days; diagnostic interval: 41 versus 22 days).

**Conclusion:**

Two-fifths of patients subsequently diagnosed with cancer have primary care blood tests as part of their diagnostic process. Given variable test use, research is needed on the clinical context in which blood tests are ordered.

## INTRODUCTION

Half of the UK population will be diagnosed with a form of cancer in their lifetime.^1^ Increasing cancer survival requires improvements in both treatment and stage at diagnosis. The latter may be achieved through earlier diagnosis of patients who are symptomatic.

The majority of patients subsequently diagnosed with cancer first present to a GP with symptoms.^2^ Decision making for managing symptomatic presentations can be guided by the results of tests carried out in primary care. In patients with symptoms of possible cancer, the diagnostic utility of abnormalities in blood test results (for example, low haemoglobin concentration, microcytosis, high platelet count, and raised inflammatory markers) has been increasingly understood in recent years.^3^^–^6

Use of common blood tests in primary care has increased over time,^7^ although how often such tests form part of care in patients who are subsequently diagnosed with cancer is unclear. Earlier research on patients with six common cancers (lung, colorectal, oesophagus, stomach, pancreas, and ovarian) indicates that between 24% (ovarian cancer) and 55% (stomach cancer) of patients had at least one blood test as part of their primary care management pre-referral.^8^ Whether there is potential for greater use of common blood tests in patients subsequently diagnosed with cancer is unclear.

Using common blood tests may represent an appealing diagnostic strategy for the large group of patients presenting with nonspecific symptoms not meeting referral criteria for specialist investigations or referrals. Patients with cancer presenting with non-specific symptoms often experience prolonged diagnostic intervals and complex care pathways.^9^^–^11

Using data on patients diagnosed in 2018 with common and rarer cancers in England, the aim in this study was to examine how often patients who are subsequently diagnosed with cancer are investigated using common blood tests in primary care as part of the management of their initial presentation and to explore the related variation in blood test use by patient characteristic, positing a priori that variation is likely by age, sex, cancer site, and symptoms, and potentially by deprivation and ethnicity.

## METHOD

### Study design and participants

Data were analysed from the National Cancer Diagnosis Audit (NCDA) 2018. The source has been described previously.^12^ Briefly, data on the diagnostic process for patients with cancer diagnosed during 2018 were collected by participating GPs based on information in the primary care records. Included patients were identified by the English Cancer Registry (National Disease Registration Service). In a previous audit, included patients were representative of the national incident population of patients with cancer, and participating practices had comparable characteristics to non- participating practices, although they were slightly larger.^12^

**Table:** How this fits in

Evidence relating to the predictive value of blood tests for cancer diagnosis is growing, yet how often they are used by GPs in patients with cancer before its diagnosis is currently unclear. In England, two-fifths of patients subsequently diagnosed with cancer in 2018 had a full blood count, urea and electrolytes, or liver function test. Blood test use was less likely in females, Black and minority ethnic, and younger patients, and more likely in those presenting with non-specific symptoms. Longer intervals to referral and diagnosis were observed in patients who were tested. This research highlights the need for interventions to obviate (in populations presenting with more specific symptoms requiring referral) and increase use (in patients presenting with less specific symptoms) of blood tests in cancer populations.

**Figure 1.**
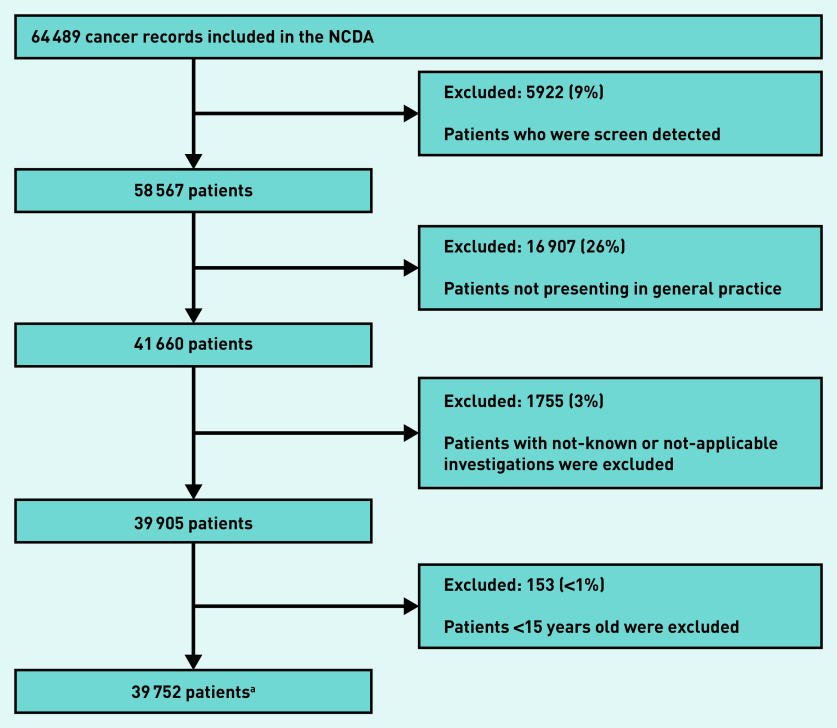
*Derivation of the analysis sample (n = 39 752).* *^a^Includes 571 patients with more than one tumour. NCDA = National Cancer Diagnosis Audit.*

The analysis sample included 39 752 patients with non-screen-detected cancer who were aged ≥15 years and who first presented in general practice and had complete information on investigation status (Figure 1).

### Outcome variable

The audit questionnaire collected information on whether blood tests were used in primary care before referral for suspected cancer, as a series of binary items: ‘Primary care led investigations that were ordered as part of the diagnostic assessment, and before referral, decided by the GP and in response to symptoms complained of, signs elicited, or abnormal test results’. In the current study common blood tests were defined as a binary variable indicating the use of at least one of: full blood count (FBC), urea and electrolytes (U&E), or liver function tests (LFTs). These blood tests were focused on as they are the ones most commonly ordered, and because abnormalities can arise from a large range of disease processes. This is unlike other, more specialist, blood tests with more specific affinity to diseases of a given body organ or system, some of which, however, were considered in addition (see below).

### Other variables

Exposure variables included: age group (15–29, 30–49, 50–69, ≥70 years), sex (male and female), ethnicity (White, Black and minority ethnic, and unknown), Index of Multiple Deprivation quintile group (based on income domain), count of pre-existing morbidities (0, 1, 2, and ≥3 conditions, and missing), cancer site (a 29-group categorical variable), and presenting symptom group.^12^

Information on presenting symptoms was collected regarding the presence of one or more of 83 pre-specified symptoms in the audit questionnaire. In the current study alarm symptoms were defined as those where the 2015 National Institute for Health and Care Excellence guidelines recommended urgent or immediate referral or specialist investigation (Supplementary Table S1).^13^ Three main groups are defined: patients presenting with alarm symptoms; those with non-alarm symptoms; and those with both alarm and non-alarm symptoms. Additionally, two further groups were considered, one group with alarm symptoms likely to indicate a medical emergency in whom primary care blood testing is not expected to be used and a group with missing information on the nature of symptoms.

The length of the primary care interval was defined consistent with the Aarhus statement: the time from first symptomatic presentation to first referral to specialist care, as was the diagnostic interval: the time from first symptomatic presentation to diagnosis, and examined by investigation status.^14^

### Analysis

Logistic regression was used to estimate crude and adjusted odds ratios (ORs) of common blood test use by age, sex, ethnicity, morbidity status, deprivation group, and symptom category. To explore whether cancer-specific factors may influence blood test use beyond these variables, in a further model, the authors additionally adjusted for cancer site. Joint Wald tests were used to assess overall variation by variable category.

The median and interquartile range (IQR) of primary care interval and diagnostic interval by test status (use/non-use of common blood tests) are described, assessing differences between symptom type groups and cancers using Kruskal–Wallis tests. To account for potential confounding or effect mediation of the observed associations between blood test use and length of the primary care interval or the diagnostic interval, quantile regression models were used, adjusting for blood test use, sex, cancer site, and symptom category (age was not included because of model non-convergence). To examine potential interactions between blood test use and symptom category, the above model was further expanded to include such an interaction term. Patients with missing information on the primary care interval (16%) and diagnostic interval (12%) were excluded from this analysis. Statistical analysis was conducted in Stata SE V.15 (StataCorp).

#### Supplementary analysis

The proportion of tested patients who received a specific common blood test or combination of tests was calculated (hereafter, patients who had a common blood test are referred to as ‘tested’ patients for brevity) and the distribution of blood tests by cancer site (Supplementary Table S2 and Table 3, respectively). Furthermore, interactions between ethnicity and deprivation were assessed within the adjusted models and no evidence for such interactions was found.

A sensitivity analysis repeated the main analysis but after excluding patients recorded as having no consultations although also recorded as having presented to their GP surgery (*n* = 2048, 5% of the main analysis sample). This group were kept in the main analysis, as a large proportion of them (*n* = 1554, 76%) were diagnosed after being referred via 2-week-wait or routinely by their GP.

## RESULTS

### Study population

Of 39 752 included patients, approximately half were ≥70 years old (49%), most were of White ethnicity (87%), and with a slight preponderance of males (55%, Table 1). Of included patients 74% had at least one chronic condition, whereas 19% had three or more. Patients were most commonly diagnosed with prostate (19%), breast (12%), or lung cancer (11%). Over one-third of patients presented with alarm symptoms alone (35%) and over two-fifths with non- alarm symptoms alone (41%), whereas less than one in five presented with both alarm and non-alarm symptoms (15%). The median primary care interval was 3 (IQR 0–20) days, and the median diagnostic interval was 39 (IQR 17–81) days.

**Table 1.** Proportions and crude/adjusted ORs examining variation in common blood test use in primary care among individuals diagnosed with cancer

**Characteristic**	**Population total, *n* (column %)**	**Received a blood test, *n* (row %)**	**Crude OR (95% CI)^a^**	***P*-value Crude for OR**	**Adjusted excluding OR^a^ cancer site (95% CI)**	***P*-value^b^ for adjusted OR excluding cancer site**	**Adjusted OR^a^ including cancer site (95% CI)**	***P*-value^b^ for adjusted OR including cancer site**
**Total**	39 752 (100)	16 427 (41)						

**Age group**				<0.001^b^		<0.001		0.001
15–29 years	553 (1)	172 (31)	0.66 (0.55 to 0.79)		0.85 (0.70 to 1.04)		0.98 (0.77 to 1.23)	
30–49 years	4009 (10)	1053 (26)	0.53 (0.49 to 0.57)		0.69 (0.63 to 0.75)		0.99 (0.90 to 1.10)	
50–69 years	15 746 (40)	6293 (40)	Ref		Ref		Ref	
≥70 years	19 444 (49)	8909 (46)	1.26 (1.21 to 1.32)		1.23 (1.18 to 1.29)		1.12 (1.06 to 1.18)	

**Sex**				<0.001		<0.001		0.009
Male	21 854 (55)	10 391 (48)	Ref		Ref		Ref	
Female	17 898 (45)	6036 (34)	0.55 (0.53 to 0.58)		0.67 (0.64 to 0.70)		0.92 (0.87 to 0.98)	

**Ethnicity^c^**				0.002		0.47		0.02
White	34 421 (87)	14 310 (42)	Ref		Ref		Ref	
Black and minority ethnic	3400 (9)	1308 (38)	0.88 (0.81 to 0.94)		0.96 (0.89 to 1.04)		0.89 (0.82 to 0.97)	
Unknown	1931 (5)	809 (42)	1.01 (0.92 to 1.11)		1.04 (0.94 to 1.15)		1.02 (0.92 to 1.14)	

**IMD**				0.22		0.10		0.11
1 — least deprived	8408 (21)	3422 (41)	Ref		Ref		Ref	
2	8222 (21)	3474 (42)	1.07 (1.00 to 1.13)		1.08 (1.01 to 1.15)		1.08 (1.01 to 1.16)	
3	7839 (20)	3219 (41)	1.02 (0.96 to 1.09)		1.04 (0.97 to 1.11)		1.01 (0.94 to 1.08)	
4	7529 (19)	3131 (42)	1.04 (0.98 to 1.11)		1.07 (1.01 to 1.15)		1.03 (0.96 to 1.11)	
5 — most deprived	7754 (20)	3181 (41)	1.01 (0.95 to 1.07)		1.04 (0.98 to 1.12)		0.99 (0.92 to 1.07)	

**Cancer^d^**				<0.001	N/A			<0.001
Leukaemia	661 (2)	552 (84)	7.69 (6.18 to 9.55)				9.24 (7.41 to 11.52)	
Myeloma	599 (2)	455 (76)	4.68 (3.84 to 5.71)				5.16 (4.22 to 6.31)	
Pancreatic	1165 (3)	826 (71)	3.61 (3.13 to 4.16)				3.52 (3.06 to 4.07)	
Liver	471 (1)	331 (70)	3.50 (2.85 to 4.31)				3.69 (2.99 to 4.55)	
Colon	2991 (8)	2093 (70)	3.47 (3.14 to 3.83)				3.84 (3.46 to 4.25)	
Stomach	727 (2)	448 (62)	2.39 (2.03 to 2.81)				2.43 (2.06 to 2.87)	
Rectal	1261 (3)	764 (61)	2.29 (2.02 to 2.61)				2.86 (2.50 to 3.28)	
CUP	629 (2)	368 (59)	2.08 (1.75 to 2.46)				2.19 (1.84 to 2.60)	
Hodgkin lymphoma	218 (<1)	121 (56)	1.83 (1.38 to 2.41)				2.27 (1.70 to 3.04)	
Ovarian	874 (2)	482 (55)	1.81 (1.56 to 2.10)				1.90 (1.63 to 2.21)	
Non-Hodgkin lymphoma	1545 (4)	852 (55)	1.82 (1.62 to 2.05)				2.15 (1.91 to 2.43)	
Kidney	969 (2)	477 (49)	1.44 (1.25 to 1.66)				1.62 (1.41 to 1.87)	
Oesophageal	1074 (3)	504 (47)	1.30 (1.13 to 1.49)				1.38 (1.20 to 1.59)	
Prostate	7499 (19)	3518 (47)	1.32 (1.23 to 1.43)				1.42 (1.31 to 1.55)	
Other	2184 (5)	1004 (46)	1.28 (1.16 to 1.42)				1.51 (1.36 to 1.68)	
Bladder	1112 (3)	481 (43)	1.13 (0.99 to 1.29)				1.33 (1.15 to 1.52)	
Mesothelioma	331 (<1)	143 (43)	1.14 (0.91 to 1.43)				1.04 (0.82 to 1.31)	
Lung	4430 (11)	1785 (40)	Ref				Ref	
Thyroid	467 (1)	179 (38)	0.95 (0.78 to 1.15)				1.38 (1.11 to 1.70)	
Brain	328 (<1)	123 (38)	0.88 (0.70 to 1.12)				0.96 (0.76 to 1.23)	
Cervical	194 (<1)	59 (30)	0.63 (0.46 to 0.87)				0.74 (0.54 to 1.03)	
Oropharynx	523 (1)	145 (28)	0.57 (0.46 to 0.70)				0.70 (0.57 to 0.86)	
Uterus	1266 (3)	318 (25)	0.49 (0.43 to 0.57)				0.65 (0.56 to 0.76)	
Larynx	297 (<1)	64 (22)	0.41 (0.31 to 0.55)				0.50 (0.37 to 0.66)	
Oral cavity	248 (<1)	28 (11)	0.18 (0.12 to 0.28)				0.26 (0.17 to 0.39)	
Testicular	340 (<1)	33 (10)	0.16 (0.11 to 0.23)				0.19 (0.13 to 0.28)	
Vulval	133 (<1)	10 (8)	0.12 (0.06 to 0.23)				0.17 (0.09 to 0.33)	
Breast	4919 (12)	209 (4)	0.07 (0.06 to 0.08)				0.09 (0.07 to 0.10)	
Melanoma	2297 (6)	55 (2)	0.04 (0.03 to 0.05)				0.05 (0.03 to 0.06)	

**Morbidities**				<0.001		0.90		0.40
0	10 145 (26)	3698 (36)	Ref		Ref		Ref	
1	12 370 (31)	5111 (41)	1.22 (1.16 to 1.29)		1.01 (0.94 to 1.06)		0.94 (0.88 to 1.01)	
2	9144 (23)	4039 (44)	1.37 (1.30 to 1.46)		1.01 (0.95 to 1.08)		0.97 (0.91 to 1.04)	
≥3	7401 (19)	3318 (45)	1.41 (1.33 to 1.50)		1.01 (0.93 to 1.07)		0.96 (0.89 to 1.03)	
Missing	692 (2)	261 (38)	N/A		N/A		N/A	

**Symptom types**				<0.001		<0.001		<0.001
Alarm only	13 778 (35)	3341 (24)	Ref		Ref		Ref	
Non-alarm only	16 487 (41)	8223 (50)	3.12 (2.97 to 3.28)		2.75 (2.61 to 2.89)		1.58 (1.49 to 1.69)	
Alarm/non-alarm	5832 (15)	3262 (56)	3.97 (3.72 to 4.23)		3.68 (3.44 to 3.93)		2.13 (1.98 to 2.30)	
Emergency only	173 (<1)	62 (36)	1.70 (1.24 to 2.34)		1.60 (1.16 to 2.21)		0.94 (0.66 to 1.32)	
Not known/not applicable	3482 (9)	1539 (44)	2.48 (2.30 to 2.69)		2.01 (1.86 to 2.18)		1.01 (0.92 to 1.10)	

a *After excluding 692 patients with missing information on morbidities, 39 060 patients remained for the logistic regression models.*

b *Post estimations using Wald tests explained the significance of the explanatory variables on predicting blood test use.*

c *The Black and minority ethnic group comprised South East Asian (n = 858, 2% of total population), Black (n = 1142, 3%), Chinese (n = 165,* <*1%), and Other (n = 1235, 3%) patients, of which 38% (n = 329), 42% (n = 474), 36% (n = 60), and 36% (n = 445) had common blood tests, respectively.*

d *Cancer site is presented in descending order of blood test use. CI = confidence interval. CUP = cancer of unknown primary. IMD = Index of Multiple Deprivation. N/A = not applicable. OR = odds ratio. Ref = reference group.*

### Use of common blood tests

A total of 16 427/39 752 (41%) patients had at least one common blood test in primary care before being diagnosed with cancer; variation in blood test use by exposure variable is described in Table 1.

Considering patient characteristics, blood test use was more frequent in males compared with females (48% versus 34%, respectively, *P*<0.001) and older patients (ranging from <32% in patients <50 years and 46% in those ≥70 years, *P*<0.001). Blood test use was less frequent in Black and minority ethnic compared with White patients (38% versus 42%, respectively, *P* = 0.002), without a clear pattern of variation by deprivation group. Use of blood tests increased with greater number of morbidities (no morbidities: 36%, ≥3 morbidities: 45%, *P*<0.001). Multivariable analysis provided concordant findings, with the exception of the association with comorbidities, which was no longer apparent.

There was very large variation in common blood test use by subsequently diagnosed cancer, ranging from 84%, 76%, and 71% in patients diagnosed with leukaemia, myeloma, and pancreatic cancer, respectively; to 8%, 4%, and 2% for patients with vulval cancer, breast cancer, and melanoma, respectively. Adjusted analyses confirmed similar patterns of variation by cancer site.

Common blood tests were used in primary care before cancer diagnosis in around half of patients presenting with either non-alarm symptoms alone or both alarm and non-alarm symptoms together (50% and 56%), but in less than a quarter (24%) of patients presenting with alarm symptoms alone. In adjusted analysis, variation by presenting symptom group remained, that is, ORs of 2.75 (95% confidence interval [CI] = 2.61 to 2.89) and 3.68 (95% CI = 3.44 to 3.93) for non-alarm symptoms alone and both alarm and non-alarm symptoms together respectively, compared with patients presenting with alarm symptoms alone. Including adjustment for cancer site attenuated these odd ratios to 1.58 (95% CI = 1.49 to 1.69) and 2.13 (95% CI = 1.98 to 2.30).

### Diagnostic timeliness by use of common blood tests

The median primary care interval was longer in patients with a blood test than those without (10 days [IQR 1–30] versus 0 day [IQR 0–13], *P* = 0.001). The median diagnostic interval was also longer in those tested than those not tested (49 days [IQR 26–95] versus 32 days [IQR 14–70], *P* = 0.001, Table 2).

**Table 2.** Median and IQR for the primary care interval and the diagnostic interval by blood test use, stratified by symptom type

**Interval**	**All patients (independently of blood test status), median (IQR) days (*n*= 37 752)**	**Patients having a common blood test, median (IQR) days (*n*= 16 427)**	**Patients not having a common blood test, median (IQR) days (*n*= 23 325)**	**Difference by common blood test use, median days**	***P*-value^a^**	**Adjusted difference in interval by common blood test use and symptom group, median (95% CI)^b^**	***P*-value**
**Primary care interval**							
Overall (*n*= 35 962)	3 (0–20)	10 (1–30)	0 (0–13)	10	<0.001	4 (3 to 5)	<0.001
Alarm only (*n*= 18 627)	0 (0–8)	4 (0–20.5)	0 (0–1)	4	<0.001	1 (1 to 1)	<0.001
Non-alarm only (*n*= 19 813)	8 (0–29)	13 (2–34)	4 (0–23)	9	<0.001	7 (6 to 8)	<0.001
Alarm/non-alarm (*n*= 5363)	2 (0–17)	6 (0–22)	0 (0–8)	6	<0.001	4 (3 to 8)	<0.001
Emergency only (*n*= 145)	0 (0–17)	9 (0–25)	0 (0–5)	9	0.017	9 (2 to 16)	0.01
Not known/not applicable (*n*= 2837)	6 (0–27)	9 (1–34)	3 (0–22)	6	<0.001	6 (4 to 8)	<0.001

**Diagnostic interval**							
Overall (*n*= 37 883)	39 (17–81)	49 (26–95)	32 (14–70)	17	<0.001	3 (1 to 5)	0.001
Alarm only (*n*= 19 190)	28 (14–61)	41 (21–79)	22 (13–51)	19	<0.001	3 (1 to 5)	<0.001
Non-alarm only (*n*= 21 478)	46 (23–91)	49 (27–97)	42 (20–85)	7	<0.001	5 (3 to 7)	<0.001
Alarm/non-alarm (*n*= 5708)	35 (16–69)	40 (21–77)	28 (14–59)	12	<0.001	3 (1 to 5)	0.007
Emergency only (*n*= 162)	42 (17–86)	51 (22–100)	37 (11–78)	14	0.57	14 (−11 to 40)	0.28
Not known/not applicable (*n*= 2872)	56 (29–107)	62 (31–117)	52 (28–100)	10	0.21	11 (6 to 16)	<0.001

a P*-value from Kruskal–Wallis test, comparing intervals in tested versus non-tested patient groups.*

b *Median (50th) quantile regression (with 500 bootstrap replications), adjusted for blood test use, symptom category, sex, cancer site, and interaction between blood test use and symptom category. Further adjusting of the diagnostic interval model for age made little difference to the findings. Adjusting of the primary care interval model for age, IMD, and comorbidities was not possible because of lack of convergence. CI = confidence interval. IMD = Index of Multiple Deprivation. IQR = interquartile range. Ref = reference group.*

Use of blood tests was associated with longer intervals across all three presenting symptom categories, although the difference was longest in patients with alarm symptoms (19 days). In adjusted analysis, substantial attenuation of the association between blood test use and the length of the primary care interval was observed (from 10 days in the observed data to 4 days, Table 2); and even more substantial attenuation of the diagnostic interval (from 17 days in the observed data to 3 days). Additional analysis indicated that the main source of these changes observed after adjustment was cancer site, that is, patients who are more likely to have a blood test are also those subsequently diagnosed with cancers associated with longer intervals. Interaction analysis further indicated that adjustment for cancer site and sex led to variable reductions in the observed differences in primary care interval between those having and those not having a blood test in patients presenting with alarm symptoms (from 4 days in the observed data to 1 day), non-alarm symptoms (from 9 days in the observed data to 7 days), or both alarm and non-alarm symptoms (from 6 days in the observed data to 4 days). A similar pattern of variable shortening of intervals by symptom category was observed for the diagnostic interval (alarm symptoms: from 19 to 3 days; non-alarm symptoms: from 7 to 5 days; alarm and non-alarm symptoms: 12 to 3 days).

### Supplementary analysis: blood test signatures and variation in use by cancer site

Among tested patients (*n* = 16 427), 95%, 89%, and 76% had FBC, U&E, and LFTs, respectively. Nearly nine in 10 (87%) of tested patients had at least two of these three blood tests and 72% had all three (Supplementary Table S2).

For six cancers (pancreatic, myeloma, liver, colon, stomach, and leukaemia) FBCs, U&E, and LFTs were ordered in over half of all patients (Table 3). Biomarker tests were most frequently used in patients diagnosed with prostate (86%) and ovarian cancer (47%) — against an average of 24% among all cancers.

**Table 3.** Table showing frequency of blood test use by cancer site^a^

**Cancer**	**Common blood tests**								**Inflammatory markers**		**Less generic tests/tests with greater affinity to specific disease processes**											
	**Any 1 of the 3 common blood tests**		**FBC**		**U&E**		**LFT**				**Cancer biomarkers,^b^ use in males**		**Cancer biomarkers,^b^ use in females**		**Serum protein**		**Ferritin**		**Bone profile**		**Amylase**	
	***n***	**%**	***n***	**%**	***n***	**%**	***n***	**%**	***n***	**%**	***n***	**%**	***n***	**%**	***n***	**%**	***n***	**%**	***n***	**%**	***n***	**%**
**Leukaemia (*n*= 661)**	552	84	543	82	370	56	340	51	216	33	39	6	5	1	46	7	115	17	117	18	9	1
**Multiple myeloma (*n*= 599)**	455	76	439	73	387	65	345	58	295	49	62	10	29	5	320	53	129	22	217	36	9	2
**Pancreas (*n*= 1165)**	826	71	790	68	763	65	773	66	488	42	89	8	110	9	45	4	224	19	239	21	194	17
**Liver (*n*= 471)**	331	70	289	61	273	58	301	64	172	37	32	7	31	7	28	6	88	19	91	19	37	8
**Colon (*n*= 2991)**	2093	70	2075	69	1751	59	1608	54	980	33	192	6	208	7	76	3	1029	34	450	15	98	3
**Stomach (*n*= 727)**	448	62	444	61	388	53	371	51	205	28	45	6	23	3	16	2	207	28	121	17	36	5
**Rectum (*n*= 1261)**	764	61	751	60	677	54	621	49	361	29	94	7	58	5	22	2	325	26	141	11	21	2
**Unknown primary (*n*= 629)**	368	59	349	55	327	52	317	50	228	36	45	7	61	10	29	5	92	15	110	17	34	5
**Ovary (*n*= 874)**	482	55	476	54	439	50	393	45	258	30	0	0	408	47	14	2	121	14	128	15	33	4
**Non-Hodgkin lymphoma (*n*= 1545)**	852	55	842	54	727	47	666	43	534	35	83	5	60	4	147	10	230	15	288	19	47	3
**Kidney (*n*= 969)**	477	49	448	46	432	45	358	37	230	24	112	12	26	3	30	3	125	13	120	12	22	2
**Oesophagus (*n*= 1074)**	504	47	496	46	456	42	422	39	230	21	36	3	18	2	19	2	190	18	125	12	37	3
**Prostate (*n*= 7499)**	3518	47	3025	40	3332	44	2337	31	1002	13	6420	86	1	<1	160	2	374	5	896	12	36	<1
**Other (*n*= 2184)**	1004	46	967	44	845	39	764	35	478	22	121	6	100	5	83	4	240	11	266	12	70	3
**Bladder (*n*= 1112)**	481	43	441	40	458	41	271	24	137	12	247	22	11	1	13	1	76	7	88	8	7	1
**Lung (*n*= 4430)**	1785	40	1720	39	1624	37	1420	32	1020	23	142	3	93	2	121	3	399	9	580	13	46	1
**Thyroid (*n*= 467)**	179	38	175	37	153	33	124	27	81	17	2	<1	7	1	4	1	21	4	37	8	0	0
**Oropharynx (*n*= 523)**	145	28	143	27	127	24	103	20	105	20	6	1	1	<1	8	2	21	4	32	6	0	0
**Uterus (*n*= 1266)**	318	25	311	25	261	21	222	18	120	9	0	0	146	12	12	1	106	8	69	5	6	<1
**Breast (*n*= 4919)**	209	4	192	4	195	4	159	3	98	2	2	<1	26	1	15	<1	42	1	76	2	8	<1
**Melanoma (*n*= 2297)**	55	2	53	2	49	2	36	2	25	1	2	<1	1	1	3	<1	11	<1	12	1	0	0
**All other cancers (*n*= 2089)^c^**	581	28	571	27	521	25	463	22	335	16	57	3	38	2	29	1	134	6	164	8	11	1
**All patients (*n*= 39 752)**	16 427	41	15 540	39	14 555	37	12 414	31	7598	19	7828	20	1461	4	1240	3	4299	11	4367	11	761	2

a *The boundaries for green–yellow–red are set at the upper, median, and lower values for each blood test. All other values are coloured proportionally.*

b *Cancer biomarkers are stratified by sex and include PSA, CEA, CA125, CA19.9, and other (unspecified).*

c *Cancer sites with* <*397 patients (that is,* <*1% of study population) were grouped together, including Hodgkin lymphoma, mesothelioma, brain, cervical, larynx, oral cavity, testicular, and vulval cancers. CA125 = cancer antigen 125. CA19.9 = cancer antigen 19-9. CEA = carcinoembryonic antigen. FBC = full blood count. U&E = urea and electrolytes. LFT = liver function test. PSA = prostate-specific antigen.*

Inflammatory marker tests were used in 19% of all patients, (*n* = 7598/39 752) and more frequently in those diagnosed with myeloma (*n* = 295/599, 49%), pancreatic cancer (*n* = 488/1165, 42%), liver cancer (*n* = 172/471, 37%), carcinoma of unknown primary (*n* = 228/629, 36%), non-Hodgkin lymphoma (*n* = 534/1545, 35%), leukaemia (*n* = 216/661, 33%), and colon cancer (*n* = 980/2991, 33%). Over half of patients diagnosed with myeloma had serum protein tests (*n* = 320/599, 53%), and over one-third had bone profile tests (*n* = 217/599, 36%). Approximately a third of patients diagnosed with colon cancer (*n* = 1029/2991, 34%) and over a quarter diagnosed with stomach cancer (*n* = 207/727, 28%) had ferritin tests, and 17% (*n* = 194/1165) of patients diagnosed with pancreatic cancer had amylase tests. Findings from the sensitivity analysis (that is, excluding patients with ‘zero’ consultations) was concordant with the main analysis (data not shown).

## DISCUSSION

### Summary

Around two-fifths of patients with cancer who presented in general practice had a common blood test as part of their diagnostic process. Patients with cancer who were female, Black and minority ethnic, or younger were less likely to have had a blood test. Use of blood tests was greater among patients presenting with less specific symptoms, although many presenting with alarm symptoms were also tested. Patients who had a blood test experienced longer diagnostic intervals *.*

### Strengths and limitations

In the current study data from a large and nationally representative sample of patients with cancer were used. The findings are based on individuals diagnosed with cancer in 2018; guidelines were updated in 2015, although mostly regarding symptom-based recommendations, not the use of blood tests.^14^

The temporal relationship of blood tests to the symptomatic presentation and consultation(s) cannot be inferred from the NCDA data. Additional chronological details on test ordering and results, as captured in routinely collected electronic healthcare (EHC) records, could have allowed more informative interpretations. From EHC records, however, it is difficult to establish the first relevant consultation with symptoms of possible cancer (and therefore harder to establish the length of diagnostic intervals). Most presenting symptoms are also under-recorded in coded data.^15^ In contrast in the NCDA, GPs could adjudicate the first relevant consultation from the patient records by accessing both structured and free-text information about presenting symptoms, thus allowing accurate estimates of diagnostic intervals.

### Comparison with existing literature

The current study expanded on previous relevant research^8^ by assessing a much larger number of cancer sites and additional factors that influence blood test use: variation by blood test type, and the impact of investigations on the diagnostic interval.

A previously published study (*n* = 100) found that over half of patients presenting in primary care with ‘unexplained’ complaints (fatigue, abdominal, and musculoskeletal complaints) were ordered blood tests.^16^ The current study found similarly high proportions of patients presenting with non-alarm symptoms having blood tests (50%). The findings concord with data on primary care investigation use in the general population, where older age was associated with a larger increase in test use over time.^7^

Common blood test use was associated with longer primary care intervals and diagnostic intervals. This suggests that GPs must balance the diagnostic utility of common blood tests against likely delays in a subsequent referral, should such a referral be required. Prolonged diagnostic intervals may encompass potentially avoidable diagnostic delays in patients with cancer.^8^^,^^17^ On the other hand, it is also possible that blood test use supports GP decisions when there is uncertainty about the underlying diagnosis; the counterfactual group in that respect is not patients in whom blood tests were not used, but patients (for example, with non-specific symptoms) in whom the intervals to diagnosis may be even longer if no blood tests were to be used. Furthermore, although an alarm symptom may not justify a referral, abnormal blood test results may provide sufficient grounds for referral even if other eligibility criteria are not fulfilled in some patients. Therefore, in some patients longer intervals associated with blood test use may be deemed acceptable for supporting the diagnostic process. The association between test use and the length of the primary care interval, and, even more so, the diagnostic interval, appeared to be partially driven by cancer site. These effects were nonetheless variable by symptom category. Differences in the length of the diagnostic intervals by blood test use chiefly relate to the post-primary care management. It is impossible to infer whether the observed differences in intervals to diagnosis represent necessary or avoidable delays in the current study, although this should be examined in further research.

### Implications for research and practice

Non-alarm symptoms have lower predictive value for cancer, likely prompting the observed greater use of blood tests in these patients. Further, patients with both alarm and non-alarm symptoms had the highest likelihood of blood testing, possibly reflecting greater degree of clinical uncertainty in these patients, or that alarm symptoms may have appeared subsequent to non-alarm symptoms. Nevertheless, half of patients presenting with non-alarm symptoms did not have a blood test. Although this cannot be directly inferred by the data in the current study, there may be greater potential for using common blood tests in these patients, particularly regarding possible referrals to rapid diagnostic centres.^6^^,^^18^^–^20

A quarter of patients who presented with alarm symptoms received a blood test before cancer diagnosis. These patients are eligible for fast-track specialist referral via the 2-week-wait pathways, yet they experience longer intervals to diagnosis, associated with the use of a blood test. Blood test use in these patients may indicate diagnostic uncertainty or situations where GPs require further diagnostic support to aid their decision making in patients not meeting all referral criteria (for example, in younger patients for those cancers with age criteria). More detailed evidence using qualitative methods would help contextualise what influences GPs’ decision making to order blood tests in patients with alarm symptoms.

Blood test use may be enhanced through interventions aimed at addressing current logistical and practical barriers (rather than decision support interventions), such as simple modifications to the choice architecture on blood test ordering forms.^21^^,^^22^

Future research should explore variation in blood test use within specific populations of patients with cancer and clinical scenarios, and incorporate qualitative methods to help understand likely drivers of use (or lack of use) of common blood tests in patients presenting to a GP with new symptoms.

In conclusion, common blood tests are frequently used in patients with cancer before referral, but their use is variable. These findings indicate potential unmet need for interventions to reduce the risk of underuse and overuse of blood tests within certain populations of patients with cancer.

## Data

Data are available through the Office of Data Release of the National Disease Registration Service of NHS Digital.
